# Absolute lymphocyte count is associated with survival in ovarian cancer independent of tumor-infiltrating lymphocytes

**DOI:** 10.1186/1479-5876-10-33

**Published:** 2012-02-27

**Authors:** Katy Milne, Cheryl Alexander, John R Webb, Winnie Sun, Kristy Dillon, Steve E Kalloger, C Blake Gilks, Blaise Clarke, Martin Köbel, Brad H Nelson

**Affiliations:** 1 Trev and Joyce Deeley Research Centre, BC Cancer Agency, Victoria, BC, Canada; 2 BC Cancer Agency, Victoria, BC, Canada; 3 Department of Biochemistry and Microbiology, University of Victoria, Victoria, BC, Canada; 4 Genetic Pathology Evaluation Centre of the Prostate Research Centre, Department of Pathology, Vancouver General Hospital, and Gynecology Tumour Group, British Columbia Cancer Agency, Vancouver, BC, Canada; 5 Department of Pathology, University of Toronto, Toronto, ON, Canada; 6 Department of Pathology and Laboratory Medicine, University of Calgary, Calgary Laboratory Services, Calgary, AB, Canada; 7 Department of Medical Genetics, University of British Columbia, Vancouver, BC, Canada

**Keywords:** Ovarian cancer, Tumor infiltrating lymphocytes, Prognosis, Tumor immunology

## Abstract

**Background:**

The immune system strongly influences outcome in patients with ovarian cancer. In particular, the absolute lymphocyte count in peripheral blood (ALC) and the presence of tumor-infiltrating lymphocytes (TIL) have each been associated with favourable prognosis. However, the mechanistic relationships between ALC, TIL and prognosis are poorly understood. We hypothesized that high ALC values might be associated with stronger tumor immunity as manifested by increased TIL, decreased tumor burden and longer survival.

**Methods:**

ALC values were collected from patient records ≥ 2 years before, during or after primary treatment for high-grade serous ovarian cancer (HGSC). Lymphocyte subsets were assessed in peripheral blood by flow cytometry. CD8+ and CD20+ TIL were assessed by immunohistochemistry.

**Results:**

Overall, patients had normal ALC values two or more years prior to diagnosis of HGSC. These values were not predictive of disease severity or survival upon subsequent development of HGSC. Rather, ALC declined upon development of HGSC in proportion to disease burden. This decline involved all lymphocyte subsets. ALC increased following surgery, remained stable during chemotherapy, but rarely recovered to pre-diagnostic levels. ALC values recorded at diagnosis did not correlate with CD8+ or CD20+ TIL but were associated with progression-free survival.

**Conclusions:**

Patients with high intrinsic ALC values show no clinical or survival advantage upon subsequent development of HGSC. ALC values at diagnosis are prognostic due to an association with disease burden rather than TIL. Therapeutic enhancement of ALC may be necessary but not sufficient to improve survival in HGSC.

## Background

Ovarian cancer affects more than 225,000 women and claims 140,000 lives world-wide each year (http://www.cancerresearchuk.org). High-grade serous epithelial ovarian cancer (HGSC) is the most common and lethal form, representing approximately two thirds of cases [1]. The large majority of HGSC cases are diagnosed at Stage III or greater, owing to the lack of effective early detection strategies [1,2]. Current standard care for advanced HGSC is cytoreductive surgery and chemotherapy with platinum-based agents (e.g., carboplatin) in combination with taxanes (e.g., paclitaxel) [2]. While most cases of HGSC are initially responsive to treatment, the majority of patients experience recurrence within 1-3 years and ultimately succumb to their disease [2]. Favorable prognostic factors for HGSC include early stage and optimal surgical de-bulking [2].

In addition to these standard prognostic factors, the host immune response to ovarian cancer has a strong influence on clinical outcomes [3,4]. In particular, the presence of CD8+ tumor-infiltrating lymphocytes (TIL) is associated with prolonged progression-free survival (PFS) and overall survival [5-9]. Accordingly, other features of cytolytic CD8+ T cell responses are also positively associated with survival, including IFN-γ, IL-12, TNF-α, the cytolytic granule component TIA-1, Major Histocompatibility Complex (MHC) class I, and others (reviewed in [4]). In addition to CD8+ T cells, CD20+ tumor-infiltrating B cells are also associated with survival in HGSC [9] and other cancers [10]. Thus, as with many other human cancers, TIL and associated factors show a clear association with clinical outcomes in ovarian cancer.

In addition to TIL, a second immunological parameter associated with outcome in ovarian cancer and other cancers is the absolute lymphocyte count (ALC), which is a measure of the number of circulating lymphocytes in peripheral blood. In healthy individuals, ALC values range from 1.0 to 4.0 Giga/L [11] and are under strong genetic control, as revealed by twin studies [12]. An individual's ALC is relatively stable through life, deviating significantly only during illness. Lymphopenia can be induced by major infection or sepsis and has strong prognostic significance in these settings [13]. Lymphopenia is also common in many autoimmune diseases, including Type 1 diabetes, rheumatoid arthritis, Sjögren's syndrome, systemic lupus erythematosus (SLE), Crohn's disease, and celiac disease [13]. In these settings, chronic lymphopenia leads to continuous homeostatic lymphocytic proliferation, which in turn may increase the likelihood of developing autoreactive lymphocytes [13]. Given the diverse array of conditions that can induce lymphopenia, it is not surprising that the Baltimore Longitudinal Study of Healthy Aging showed that low ALC predicted death within 3 years from any cause [14]. Thus, despite being a relatively crude measure, ALC serves as a useful barometer of immune function and general health in humans.

Cancer patients also frequently show decreased ALC values at diagnosis (reviewed in [15]). In 1970, Riesco reported that ALC was positively associated with the "curability" of a variety of cancers [16]. Similar associations between ALC and survival have been reported for a wide variety of epithelial, connective tissue and lymphoid cancers [17], including ovarian cancer [18,19]. In addition to survival, ALC has been associated with response to various cancer treatments, including chemotherapy [20] and autologous hematopoietic stem cell transplantation for hematopoietic [15] and epithelial cancers [21,22].

Many researchers have speculated that ALC might influence cancer outcomes through an immunological mechanism. Indeed, in Riesco's 1970 paper, he noted that "these findings agree with the idea of the participation of lymphocytes in the mechanism of the eventual phenomenon of anticancerous immunity" [16]. If so, then it might be beneficial to attempt to increase ALC in patients as a means to promote tumor immunity. Importantly, however, this concept has not been critically evaluated. It has yet to be determined whether ALC is a marker versus mediator of favorable prognosis. To address this issue, we investigated the relationship between ALC, TIL and outcome in HGSC. Specifically, we hypothesized that patients with high ALC values might mount stronger immune responses against their tumor, resulting in increased TIL and prolonged survival. To gain insight into the causal relationship between ALC and prognosis, we considered not only ALC values recorded at the time of cancer diagnosis, but also intrinsic ALC values recorded two or more years prior to diagnosis.

## Methods

### Patients, clinical data and immunological data

This study was approved by the Research Ethics Board of the British Columbia Cancer Agency (BCCA) and University of British Columbia. Patients were admitted to the BCCA with high-grade serous epithelial ovarian cancer (HGSC) between January 2005 and January 2010. Inclusion criteria included (a) availability of ALC data, and (b) treatment with primary cytoreductive surgery followed by chemotherapy with carboplatin with or without paclitaxel. Exclusion criteria included previous or concurrent cancers, neoadjuvant chemotherapy, and delivery of radiation therapy as part of first line treatment. Clinical data including ALC values were obtained from the BCCA's Oncology Reporting System and Cancer Agency Information System under the Research Ethics Board approval obtained by the Cheryl Brown Ovarian Cancer Outcomes Unit, which permits access to medical records related to treatment of all ovarian cancer patients admitted to the BCCA. Pre-diagnostic ALC values were obtained from primary care physicians for 113 HGSC patients (Cohort A, Table 1). Progression-free survival was defined as the time from diagnosis to first recurrence as defined by a combination of physical findings, radiological evidence or increased CA125. Of the 113 HGSC patients for whom pre-diagnostic ALC values were available, 80 also had pre-treatment ALC values available for comparison.

**Table 1:** Clinicopathological features of patient cohorts.

Cohort A		Cohort B	
Total number of patients	113	Total number of patients	110

Age at surgery (years)		Age at Surgery (years)	

Mean	64.4	Mean	59.2

Standard deviation	10.4	Standard deviation	10.8

Range	40-88	Range	35-83

Median	65.5	Median	58.5

Progression-free survival (years)		Disease-specific survival (years)	

Mean	1.5	Mean	4.3

Standard deviation	1.0	Standard deviation	2.3

Range	0.1-5.0	Range	0.6-10.8

Median	1.2	Median	3.9

Grade		Grade	

1	0	1	0

2	4	2	7

3	97	3	98

2 or 3 (unspecified)	12	2 or 3 (unspecified)	5

Stage		Stage	

I	7	I	8

II	15	II	10

III	81	III	90

IV	7	IV	2

Unknown	3	Unknown	0

Cohort A includes patients for whom pre-diagnostic ALC values were available. Cohort B includes patients for whom both ALC values and CD8+ and CD20+ TIL scores were available

The flow cytometry study involved blood samples collected prospectively from 15 HGSC patients and 16 age-matched cancer-free females. The TIL study involved 110 HGSC patients (Cohort B, Table 1) for whom mid-chemotherapy ALC values were available and primary tumor specimens had previously been stained and scored for CD8+ and CD20+ TIL [7,9,23]. Cohorts A and B were largely independent of each other, except for an overlap of 18 patients.

### Analysis of lymphocyte subsets by flow cytometry

PBMCs were stained for 30 min at room temperature with the following antibodies: CD3-FITC/PECy7/APC (clone HIT3a), CD4-APCH7 (clone RPA-T4), CD8-PerCP (clone RPA-T8), CD45RO-PE (clone UCHL1), CD45RA-APC (clone HI100), CD19-PE (clone SJ25C1), CD20-FITC (clone 2H7), CD25-FITC (clone M-A251), CD56-APC (clone B159), CD14APCH7 (clone M5E2) (all from BD Pharmingen Canada, Mississauga, ON). FoxP3 was detected by intracellular staining following the manufacturer's instructions (FoxP3-PE, clone PCH101, eBioscience, San Diego, CA). Samples were analyzed on a BD Influx multi-channel flow cytometer. Data analysis was performed using FlowJo software.

### Statistical analysis

Statistical analysis was performed with GraphPad Prism 5.0 and included log-rank test for survival analysis; generation of Kaplan-Meier curves; Kruskal Wallis test; Friedman ANOVA; Dunn's post test; Mann-Whitney U test; and Wilcoxon Signed Rank test, as indicated in the text.

## Results

### ALC declines upon the development of ovarian cancer

To assess whether individuals with high intrinsic ALC values might experience a more favorable prognosis upon the development of HGSC, we studied a cohort of HGSC patients for whom we obtained matched ALC values recorded ≥ 2 years prior to diagnosis (referred to as "pre-diagnostic ALC") and at the time of diagnosis before any form of treatment (referred to as "pre-treatment ALC"). All patients went on to receive standard treatment consisting of primary cytoreductive surgery followed by platinum- and taxane-based chemotherapy.

**Figure 1:**
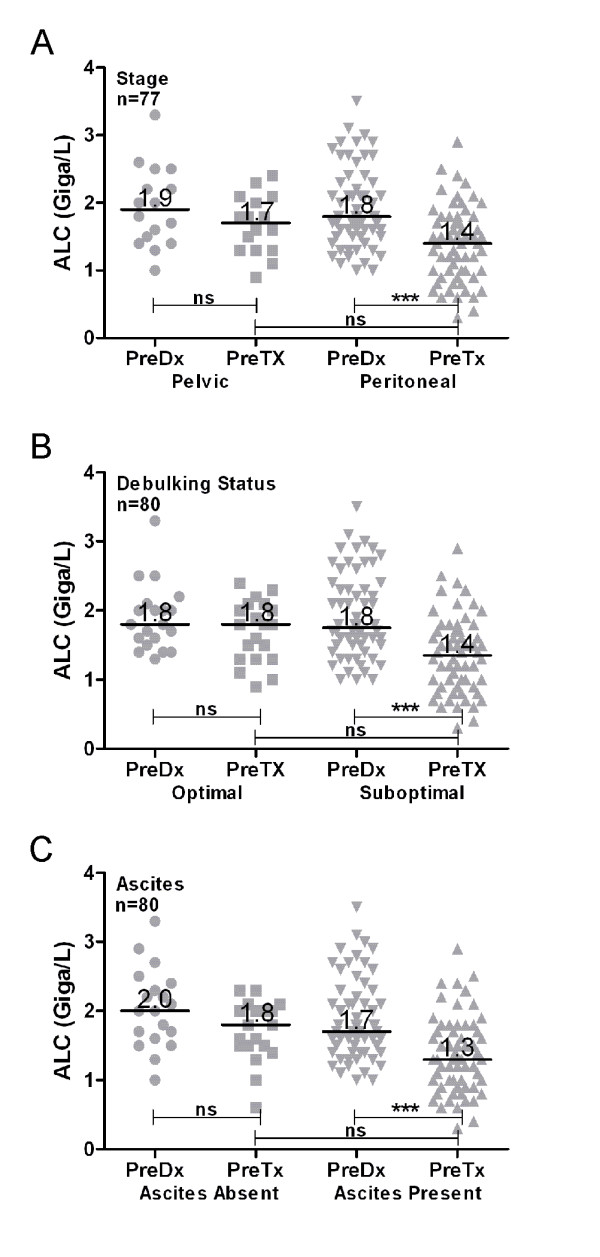
**ALC declines upon development of HGSC in proportion to disease burden**. Change in ALC from pre-diagnostic (preDx) to pre-treatment (preTx) levels according to **(A) **stage of disease (pelvic involvement encompasses Stage I and II disease, peritoneal involvement is seen in Stage III and IV patients), **(B) **surgical debulking status (no visible residual vs visible residual), or **(C) **presence of ascites. Statistical significance measured by Kruskal-Wallis ANOVA and Dunn's post test.

The mean pre-diagnostic ALC was 1.9 Giga/L, which is consistent with the mean for healthy women (1.9-1.95 Giga/L) [11]. This indicates that HGSC patients have, on average, normal ALC values prior to the clinical presentation of cancer. By contrast, the mean pre-treatment ALC was significantly lower at 1.4 Giga/L (*p *< 0.0001 by Wilcoxon signed rank test), indicating that the development of HGSC is associated with a marked decline in ALC. The extent of ALC decline was proportionate to disease severity. For example, advanced stage patients underwent a greater decline in ALC than early stage patients (median from 1.8 to 1.4 vs. 1.9 to 1.7 Giga/L; Figure 1A). Likewise, patients who after primary surgery were classified as having suboptimally debulked disease (defined as any visible residual) had a lower pre-treatment ALC than optimally debulked patients (defined as no visible residual) (median from 1.8 to 1.4 vs. 1.8 to 1.8 Giga/L; Figure 1B). Similar results were seen when patients were stratified according to the presence of ascites (Figure 1C). Note that these relationships were not causally related to treatment, since pre-treatment ALC was recorded prior to surgery or chemotherapy. Rather, the results indicate that patients with higher disease burden experience a greater decline in ALC from their pre-diagnostic values.

### ALC and lymphocyte subsets

**Figure 2:**
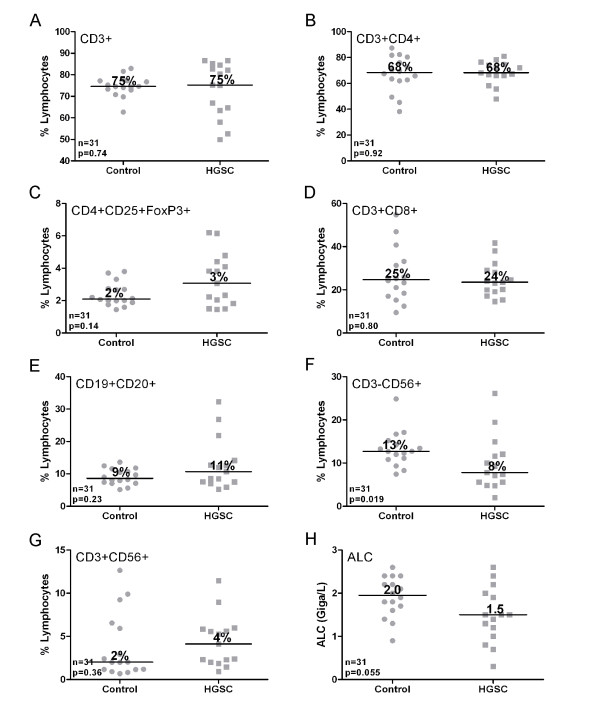
**Flow cytometric analysis of lymphocyte subsets in blood from controls and HGSC patients in comparison to ALC**. PBMCs from controls and cancer patients (pre-treatment) were stained for the indicated markers and run on a BD Influx Multi-channel Flow Cytometer. Mann-Whitney U tests were performed to examine the differences between controls and cancer patients with respect to **(A) **CD3+, **(B) **CD3 + CD4+, **(C) **CD4 + CD25 + FoxP3+, **(D) **CD3+ CD8+, **(E) **CD19 + CD20+, **(F) **CD3-CD56 + and **(G) **CD3+CD56 + lymphocytes and **(H) **ALC values for each group.

To determine which lymphocyte subsets are reduced in patients with low ALC values, we used flow cytometry to analyze peripheral blood lymphocytes collected prior to treatment from 15 HGSC patients as well as 16 age-matched females with no personal history of cancer. As expected, cancer patients trended toward a lower median ALC than controls (1.5 vs. 2.0 Giga/L), although this did not reach significance by Mann-Whitney test (*p *= 0.055) (Figure 2). Nevertheless, patients showed normal mean percentages of CD3+ T cells, CD3 + CD4+ T cells, CD3 + CD8+ T cells, CD3+ CD4 + CD25+ FoxP3+ Treg-like cells, CD3 + CD56+ NKT-like cells, and CD19 + CD20+ B cells (Figure 2). The only statistically significant difference was that cancer patients had a modestly decreased percentage of NK-like cells (CD3-CD56+) compared to controls (8% vs. 13% respectively, *p *= 0.019 by Mann-Whitney test). Thus, there were no gross deficiencies in lymphocyte subsets in HGSC patients.

### ALC and prognosis

**Figure 3:**
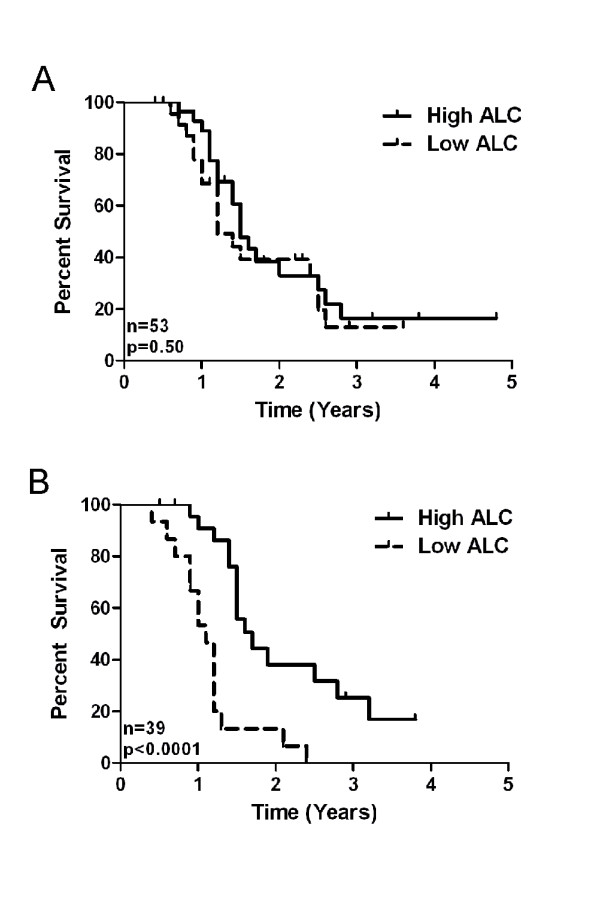
**Relationship between ALC and clinical outcome in ovarian cancer**. HGSC patients were stratified into high and low quartiles based on ALC values recorded **(A) **≤ 2 years prior to HGSC diagnosis, or **(B) **just prior to treatment. Kaplan Meier analysis was performed to compare PFS between groups; significance was assessed with the log-rank test. N values represent the number of patients in the highest and lowest quartiles only, the middle 2 quartiles have been omitted; panel B has a higher n value as not all patients with pre-diagnostic ALCs available had pre-treatment values available.

We examined the relationship between ALC and progression-free survival (PFS) by stratifying patients into quartiles based on their pre-diagnostic or pre-treatment ALC values. For pre-diagnostic ALC values, patients in the highest quartile showed equivalent PFS to patients in the lowest quartile (*p *= 0.50; Figure 3A). By contrast, for pre-treatment ALC values, patients in the highest quartile (ALC ≥ 1.8 Giga/L) showed a mean PFS of 1.7 years compared to 1.0 years for patients in the lowest quartile (ALC ≤ 1.0) (*p *< 0.0001) (Figure 3B). Thus, HGSC patients with high intrinsic ALC values show no apparent survival advantage upon the development of HGSC. Rather, patients with high ALC values at the time of diagnosis have a significantly longer PFS.

### Effect of standard treatment on ALC

**Figure 4:**
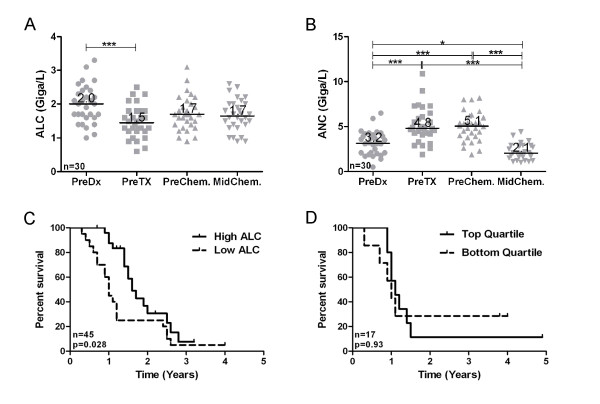
**Effect of surgery and chemotherapy on ALC and ANC and relationship to clinical outcome**. Friedman test followed by Dunn's post test was used to analyze **(A) **ALC and **(B) **ANC values collected prior to diagnosis (PreDx), prior to surgical treatment (PreTx), immediately prior to chemotherapy (PreChem) and immediately prior to the 4th cycle of chemotherapy (MidChem). For ALC, only the change from the pre-diagnostic time point to pre-treatment was significant. For ANC, the only insignificant change was from the pre-treatment to pre-chemotherapy time points. **(C) **ALC values recorded at the mid point of chemotherapy are associated with prolonged PFS by Kaplan-Meier analysis. **(D) **Extent of ALC recovery at the mid-point of chemotherapy (relative to pre-diagnostic ALC) is not associated with PFS in suboptimally debulked patients by Kaplan-Meier analysis.

The preceding analyses indicated that patients with the highest disease burden experienced the greatest decline in ALC from pre-diagnostic to pre-treatment timepoints (Figure 1). We therefore reasoned that surgery and chemotherapy, by reducing disease burden, might promote recovery of ALC. To address this possibility, we assessed ALC values in the subset of patients for whom ALC data was available across four clinical time points: pre-diagnostic, pre-treatment (i.e., prior to primary surgery or chemotherapy), pre-chemotherapy (i.e., post-surgery but prior to chemotherapy), and at the mid point of chemotherapy (pre-cycle 4). Similar to the preceding results, the median pre-diagnostic ALC was 2.0 Giga/L and declined to a pre-treatment median of 1.5 Giga/L (Figure 4A). The median ALC increased to 1.7 Giga/L after primary surgery and was maintained at 1.7 Giga/L during chemotherapy (Figure 4A).

For comparison, we also evaluated changes in neutrophils. In contrast to ALC, the median absolute neutrophil count (ANC) increased from pre-diagnostic to pre-treatment time points (3.2 vs. 4.8 Giga/L). The mean ANC did not change significantly after surgery but dropped sharply during chemotherapy (from 5.1 to 2.1 Giga/L) (Figure 4B), which is a common clinical observation.

ALC values are not routinely recorded at our institution once standard treatment is completed, therefore it was difficult to consistently obtain ALC values during the post-treatment phase. Nonetheless, in the subset of patients who were in remission > 1 year after surgery, the mean ALC remained low at 1.5 Giga/L. In the subset of patients who succumbed to their disease, ALC declined dramatically to 1.0 Giga/L within a year of death (data not shown).

The preceding results support the notion that standard treatment, by reducing disease burden, promotes recovery of ALC, although rarely to pre-diagnostic levels. We investigated whether the extent of ALC recovery might be associated with prognosis. To this end, we first assessed ALC values recorded during chemotherapy, since this was the time point at which most patients achieved maximal ALC recovery. Indeed, ALC values recorded at the midpoint of chemotherapy were strongly associated with PFS (Figure 4C). However, this analysis did not measure ALC recovery *per se*, since it included patients with low disease burden who had experienced only minimal ALC decline at the time of diagnosis. To reduce this confounding effect, we analyzed ALC recovery in suboptimally de-bulked patients. As mentioned, this subgroup experienced the greatest decline in ALC from pre-diagnostic to pre-treatment levels (Figure 1B). During treatment, this subgroup showed a wide range of ALC recovery, from 48% to 143% (data not shown). This indicates that even among patients with high-risk disease, some undergo a more robust immune recovery than others. Unexpectedly, however, the extent of ALC recovery showed no association with PFS (Figure 4D). This suggests that restoration of normal lymphocyte levels during treatment does not confer a survival advantage.

### ALC and tumor-infiltrating lymphocytes

**Figure 5:**
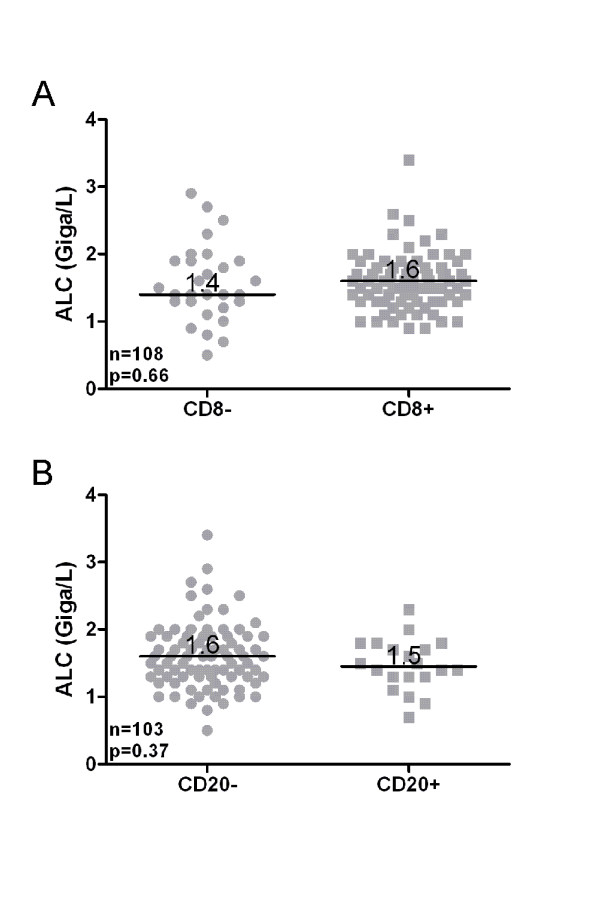
**Relationship between ALC and (A) CD8+ and (B) CD20+ TIL**. Tumors were scored as positive or negative for the indicated TIL subpopulations. Average ALC values recorded during chemotherapy were compared by Mann-Whitney U test.

Collectively, our results indicate that ALC values recorded at diagnosis or during treatment have strong prognostic significance. To address whether this association might have an immunological basis, we investigated the relationship between ALC and TIL in a cohort of 110 HGSC patients for whom primary tumor specimens were available. For this analysis, we used the average of the ALC values recorded during chemotherapy, since this was the most consistently available time point in this patient cohort. We focused on CD8+ and CD20+ TIL, since these lymphocyte subsets showed the strongest association with survival in our previous TIL study [9]. Primary tumor samples were stained with antibodies to CD8 and CD20, and cases were scored as positive or negative for TIL according to established criteria [7,9]. Consistent with our prior report [9], a significant proportion of tumors were positive for intraepithelial CD8+ TIL (73.1% 79/108), and a smaller proportion contained intraepithelial CD20+ TIL (19.4% 20/103). ALC values showed no association with the presence of intraepithelial CD8+ TIL (*p *= 0.66, Mann-Whitney) or CD20+ TIL (*p *= 0.37, Mann-Whitney) (Figure 5). This was true for the entire cohort (Figure 5) and after stratification according to surgical de-bulking status (data not shown). Similar results were seen using pre-treatment ALC values, although the sample size was smaller due to the limited availability of pre-treatment ALC values in this cohort (data not shown). Thus, ALC and TIL appear to be independent parameters that are both associated with outcome in HGSC.

## Discussion

ALC is associated with survival in ovarian cancer and many other cancers, but the mechanistic basis of this association is poorly understood. We hypothesized that HGSC patients with high intrinsic ALC values might mount stronger anti-tumor immune responses, reflected by increased TIL, decreased tumor burden and prolonged survival. Contrary to this hypothesis, we found that intrinsic ALC values (measured ≥ 2 years prior to the diagnosis of cancer) have no bearing on tumor burden or progression-free survival upon the subsequent development of HGSC. Instead, we found that ALC declines upon development of HGSC to levels proportionate to tumor burden. Accordingly, ALC values recorded at the time of diagnosis are strongly associated with prognosis. ALC values increase after cytoreductive surgery but rarely return to intrinsic levels, indicating long-term impairment of lymphoid homeostasis. Finally, we showed that ALC is not associated with the presence of CD8+ or CD20+ TIL. Collectively, our findings demonstrate that ALC and TIL are independent immunological parameters associated with outcome in HGSC. In the case of ALC, this appears to reflect an association with disease burden rather than an immunological mechanism.

To determine patients' intrinsic ALC, we obtained values that were recorded two or more years prior to HGSC diagnosis. While the natural history of HGSC remains poorly understood, the latency between disease initiation and clinical diagnosis is estimated to be 3-4 years based on biomarker and modeling studies [24,25]. Thus, with a two-year threshold, some patients in our cohort presumably harbored small occult cancers at the time their pre-diagnostic ALC values were measured. Nevertheless, we note that the mean pre-diagnostic ALC for our cohort (1.9 Giga/L) was similar to the mean for healthy women (1.9-1.95 Giga/L) [11] and in most cases did not decline until ≤ 1 year prior to HGSC diagnosis. A related caveat is that pre-diagnostic ALC values might have been collected for reasons such as acute illness (e.g., infection) and hence might not reflect "intrinsic" ALC values in all cases. That said, for several patients we were able to obtain serial ALC values over several years. In general, these values were stable over time until declining ≤ 1 year prior to HGSC diagnosis.

What mechanism(s) might underlie the decline in ALC upon the development of HGSC or other cancers? Similar to the development of lymphopenia during sepsis, lower ALC values might reflect a response to systemic inflammation. Ovarian cancer is often accompanied by systemic inflammation, as evidenced by increased neutrophil counts (Figure 4). Moreover, several markers of inflammation have been associated with increased tumor burden and/or adverse outcome in ovarian cancer, including high neutrophil-to-lymphocyte ratio [19]; high monocyte count [26]; elevated C-reactive protein and hypoalbuminaemia [27]; and elevated IL-6 and IL-8 levels in ascites fluid [28,29]. Inflammation could depress ALC values by several possible mechanisms. In mice, IL-6 has been shown to induce Id1 expression in uncommitted hematopoietic progenitors, thereby promoting myelopoiesis over lymphopoiesis [30]. Consistent with this, lymphopenia correlates strongly with increased serum levels of IL-6, as well as soluble IL-2 receptor and TNF receptor in soft tissue sarcomas [31]. In addition to inflammatory cytokines, ovarian cancer and other carcinomas are also associated with elevated levels of Vascular Endothelial Growth Factor [32], which can inhibit T cell development [33].

ALC might also decline due to apoptosis of lymphocytes. For example, during sepsis, lymphopenia is associated with apoptosis-induced depletion of lymphocytes and dendritic cells [34]. Lymphopenia has also been attributed to lymphocyte apoptosis in pancreatitis [35] and measles infection [36]. Finally, CD8+ T cells from cancer patients have been shown to undergo apoptosis in response to tumor-derived microvesicles expressing tumor antigens, Fas ligand and MHC class I [37]. In summary, the decline in ALC observed in cancer patients may reflect both reduced production and increased apoptosis of lymphoyctes. It is noteworthy that ALC rarely recovers to pre-diagnostic levels in HGSC (Figure 4 and data not shown). Similar results were seen in head and neck cancer, even in patients with no evidence of disease two or more years after treatment [38]. Thus, cancer can lead to the long-term impairment of lymphoid homeostasis, a condition that may need to be addressed for immunotherapy to be effective.

In contrast to our initial hypothesis, we failed to find an association between ALC and TIL (Figure 5). Although we only evaluated CD8+ and CD20+ TIL, these two subsets are strongly associated with patient survival and hence are most relevant to our hypothesis [4-9]. Whether ALC values are associated with other mechanisms of anti-tumor immunity, such as innate or humoral responses, remains to be determined. Even so, these other immune mechanisms have yet to show the same prognostic significance as TIL. As for the issue of why TIL densities vary among patients, previous work in ovarian cancer has linked the presence of TIL to chemokine profiles in tumors [3], functional status of the BRCA DNA repair pathway [7], and other factors (reviewed in [4]). It appears these mechanisms have a greater influence than ALC in regulating TIL responses.

Our results have implications for cancer immunotherapy, in particular strategies designed to non-specifically increase lymphocyte numbers in cancer patients [39]. For example, administration of IL-2 causes transient lymphocytosis (increased ALC), which correlates with tumor response in metastatic melanoma [40] and renal cancer [41]. Administration of IL-2 prior to surgery can prevent lymphopenia and provide possible survival benefit in pancreatic [42], colorectal [43], and gastric cancer [44]. In the latter study, IL-2 administration increased both ALC and TIL, suggesting these two immune parameters can be enhanced together. IL-7 can also be used to increase circulating lymphocytes and has the advantage of being less toxic than IL-2. For example, cancer patients treated with IL-7 experienced marked increases in peripheral CD4+ and CD8+ T cells, resulting in a rejuvenated circulating T-cell profile [45]. Finally, ALC can also be increased through cell therapy. In the setting of autologous hematopoietic stem cell transplantation (AHSCT), the extent of ALC or T cell recovery has been positively associated with survival in myeloma and lymphoma, breast cancer, and ovarian cancer [15,21,22]. Based on these results, researchers at the Mayo Clinic are investigating whether increasing the lymphocyte content of the AHSCT cell product can improve clinical outcomes in lymphoma [7,15].

Although the above approaches may have merit, it is noteworthy that in the present study the extent of ALC recovery after standard treatment had no bearing on prognosis (at least in suboptimally de-bulked patients), suggesting that enhancing ALC alone does not confer survival benefit. This implies that interventions that simply increase ALC may not be sufficient to elicit effective tumor immunity. A more promising goal may be to develop strategies that not only increase ALC but, in the process, skew lymphocyte recovery in favour of a tumor-reactive repertoire.

## Conclusions

Patients with high intrinsic ALC values showed no advantage with respect to disease severity or progression-free survival upon development of HGSC. ALC values recorded at the time of diagnosis were strongly associated with disease burden and thus prognosis. ALC values were not associated with the presence of TIL. From a therapeutic perspective, our results suggest that simply increasing ALC may not be sufficient to promote clinically significant antitumor responses.

## Abbreviations

ALC: Absolute lymphocyte count; ANC: Absolute neutrophils count; HGSC: High grade serous carcinoma; TIL: Tumor infiltrating lymphocytes; PFS: Progression free survival; AHSCT: Autologous hematopoietic stem cell transplantation.

## Competing interests

The authors declare that they have no competing interests.

## Authors' contributions

KM conceived the study and its design, acquired clinical data, analysed the data and drafted the manuscript. CA acquired clinical data. JRW performed FACS experiments and analysis. WS performed FACS experiments and analysis. KD acquired and processed control blood and coordinated the acquisition of WBC differentials. SEK contributed access to clinical information associated with TMAs used and performed statistical analysis. CBG provided access to TMAs for analysis. BC scored immunohistochemical staining. MK scored immunohistochemical staining. BNH conceived the study and its design and drafted the manuscript. All authors contributed to the revision of the manuscript and approved the final manuscript.
